# Assessment of Peste des Petits Ruminants Antibodies in Vaccinated Pregnant Ewes of Kazakh Breed Fine-Fleeced and Determination of the Decreasing Trend of Maternal Immunity in Their Lambs

**DOI:** 10.3390/v15102054

**Published:** 2023-10-06

**Authors:** Zhanat Amanova, Sholpan Turyskeldy, Zhanat Kondybaeva, Zhanna Sametova, Abdurakhman Usembai, Aslan Kerimbayev, Yerbol Bulatov

**Affiliations:** Research Institute for Biological Safety Problems, Gvardeiskiy 080409, Kazakhstan; smankizi@biosafety.kz (S.T.); zh.kondybaeva@biosafety.kz (Z.K.); zh.sametova@biosafety.kz (Z.S.); a.ussenbay@biosafety.kz (A.U.); a.kerimbayev@biosafety.kz (A.K.); ye.bulatov@biosafety.kz (Y.B.)

**Keywords:** peste des petits ruminants, ewes, lambs, vaccination, passive immunity

## Abstract

In this article, we first assessed peste des petits ruminants (PPR) antibodies in vaccinated pregnant ewes of Kazakh breed fine-fleeced immunized with the PPR vaccine and the duration of maternal immunity in their lambs. Ewes in the last trimester of pregnancy and gestation were immunized with a vaccine from the Nigeria 75/1 strain of the PPR virus (PPRV) produced by the Research Institute of Biological Safety Problems (RIBSP), Kazakhstan. Serum samples from lambs born from vaccinated and unvaccinated ewes were collected a week after birth and at intervals of 7 days for 18 weeks after birth. Serum samples collected from lambs were tested for PPR antibodies using competitive ELISA and virus neutralization test (VNT). Maternal antibodies (MAs) in lambs born from vaccinated ewes were detected for up to 18 weeks, with a tendency to decrease starting at week 14, and by the end of the experiment receded below the protective level (<1:8). In the blood serum of a 14-week-old lamb with MAs (1:8), post vaccination with a field dose (10^3^ TCID50) of the vaccine against PPR, the titers of protective antibodies against PPRV increased to 1:16 on day 14 post vaccination, and the lamb was protected from infection with the field PPRV. A lamb of the same age with MAs in the 1:8 titer was 100% protected from infection with the field PPRV. Therefore, it is recommended that lambs of the Kazakh fine-wool breed be immunized from the age of 14 weeks or older to avoid a period of susceptibility.

## 1. Introduction

Peste des petits ruminants (PPR) is a highly contagious, infectious viral disease of small ruminant species that is caused by the peste des petits ruminants virus (PPRV), the prototype member of the *Morbillivirus* genus in the Paramyxoviridae family [1]. The disease is currently endemic in most of Africa, the Middle East, South Asia and China [2]. Despite strict control measures including statutory regulations along with the availability of vaccines and diagnostics, PPR remains a constant threat [3]. Currently, PPR is not an endemic disease for the Republic of Kazakhstan. However, it should be noted that the results obtained by Lundervold et al. in 1997–1998, when conducting a study on the detection of antibodies in sheep, goats and cattle in Central Kazakhstan, showed that PPRV could circulate in the country unnoticed [4]. Five years later, in 2003, an outbreak of PPR occurred in the Turkestan region in small ruminant livestock [4,5]. Since then, until the end of 2014, the OIE has not received official reports of cases of infection with the PPRV from Kazakhstan. However, more than 10 years later, PPR caused three outbreaks in individual farms at the end of 2014 in Southern Kazakhstan (Zhambyl region). Based on partial N gene sequencing, the identified strains showed high similarity to PPRV strains from China from 2013/2014, suggesting PPR transboundary spread between the two countries. The three outbreaks did not have any obvious epidemiological linkages, suggesting that PPRV may have been persistently present at a subclinical level despite vaccination efforts [6,7]. These data are of the greatest concern because Kazakhstan is home to the world’s three largest populations of *Saiga* antelope, which are susceptible to the PPR virus. However, a serological study conducted in Kazakhstan in the period from 2012 to 2014 did not reveal *Saiga* antelopes seropositive for PPR [6].

To date, due to the outbreak of PPR in Mongolia, the regions of Eastern Kazakhstan are at risk of PPR infection.

To achieve PPR eradication, which is targeted for 2030, a PPR Global Control and Eradication Strategy (PPR GCES) was developed, based on a progressive reduction in PPR incidence and spread through targeted vaccination [8].

Within the territory of the Republic of Kazakhstan, annual routine vaccination of small ruminants against PPR is carried out in border regions in order to prevent the introduction of PPR from neighboring countries that are disadvantaged by this disease. In Kazakhstan, live attenuated vaccines are mainly used for the prevention of PPR, which create 1-year immunity against PPR.

To increase the immunogenicity of the PPR vaccine, we developed a live vaccine based on the Nigeria/75/1 strain. The live vaccine from the Nigeria/75/1 strain is the most widely used PPR vaccine approved by the OIE [9]. It has been reported that the Nigeria 75/1 strain of the PPRV causes persistent immunity in once-immunized animals for up to 3 years [10].

There are few reports on the duration of persistence of maternal antibodies in lambs/kids born from ewes vaccinated against PPR [11]. Maternal antibodies transmitted to lambs from vaccinated ewes through colostrum protect newborn lambs from infection for a certain period. However, maternal antibodies can negatively affect the effectiveness of vaccination with live vaccines. Therefore, before starting vaccination, it is recommended to check the immunological status of lambs.

To avoid negative consequences when immunizing young lambs and to determine the optimal age of lambs for vaccination against PPR, we studied the persistence of maternal antibodies in lambs born from ewes of Kazakh breed fine-fleeced vaccinated against PPR.

## 2. Materials and Methods

### 2.1. Animals

In the southern regions of Kazakhstan, sheep mating occurs more often in the months of October and November, and lambing occurs most often in March and April. In this regard, in September, we purchased sheep of Kazakh breed fine-fleeced of either sex (3 males and 15 females aged 12–12.5 months). All animals were dewormed by oral administration of albendazole. Food and water were available in unlimited quantities throughout the experiment. The animals were labeled and kept in isolation in the RIBSP quarantine zone for 30 days. Body temperature was regularly measured in the animals, and blood serum was collected to determine the presence of specific antibodies to PPRV. Detection of antibodies to PPRV in sheep sera was performed using a virus neutralization test (VNT) [12]. The animals were not found to have specific antibodies to the PPRV, and they had not previously been vaccinated against this disease.

Ewes in the third trimester of pregnancy were used for the research. Pregnancy periods in ewes were determined using ultrasonography. Estrus synchronization in ewes was not performed; therefore, as soon as the lambs were born, they were included in experimental studies. Newborn lambs were kept together with their mothers so that they could suck colostrum freely.

Experimental studies were conducted in compliance with international and national ethical standards. The protocol was approved by the Ethics Committee for Animal Experimentation at the RIBSP (Permission number: 2908/22).

### 2.2. Vaccination

Out of 15 females, 10 became pregnant. Ewes (n = 6) in the 3rd trimester of pregnancy were immunized with PPR vaccine (Nigeria 75/1 strain) produced by the RIBSP, Kazakhstan. Ewes were immunized subcutaneously with a single field dose of the vaccine (1.0 × 10^3^ TCID 50/mL). Four healthy ewes in the 3rd trimester of pregnancy were used as control animals (unvaccinated). All of the vaccinated and unvaccinated ewes were monitored daily for clinical signs of PPR, and rectal temperature was measured for 21 days post vaccination (dpv).

### 2.3. Blood Sample Collection

Blood samples of vaccinated and control ewes taken on days 0, 7, 14 and 21 were tested for the presence of antibodies in blood sera. Blood samples were taken from newborn lambs at the age of 1 week with an interval of 7 days for 18 weeks. The obtained blood sera of experimental animals were inactivated at a temperature of 56 °C for 30 min and placed in freezers with a storage temperature of −20 °C.

### 2.4. Serology Tests

#### 2.4.1. VNT

PPRV antibodies were detected using VNT [12] and ELISA [13]. The titer of virus neutralizing antibodies (VNA) was calculated by the Reed and Muench method [14]. The viral neutralizing activity of the serum was expressed in the neutralization index, which is the difference in the logarithms of the titer of the virus in the presence of specific and normal serum.

The highest serum dilution was taken as the antibody titer, which was able to suppress the activity of the virus injected at the specified dose in 50% of the infected culture.

VNT was carried out in two repetitions, and the average value of the two tests was used when analyzing the results of the study.

#### 2.4.2. Competitive ELISA (c-ELISA)

Blood sera of experimental animals were additionally examined for the presence of antibodies in c-ELISA. As an additional test for detecting anti-PPRV antibodies to nucleoprotein (NP) in the blood sera of experimental animals, a c-ELISA kit (ID Screen^®^PPR Competition (PPRC-4P), ID.vet, Montpellier, France) was used [13]. The ELISA test was performed according to the manufacturer’s instructions. The results of the ELISA analysis were read using a spectrophotometer at a wavelength of 450 nm. The test results were considered reliable if the average value of the ODc− > 0.7 and the ratio of the average values of the ODc+/ODc− < 0.3. The S/N percentage (S/N%) value was calculated for each sample using the formula sample OD/ODc− ×100%. Samples showing a ratio of S/N% = 50% were considered positive, whereas S/N > 60% were considered negative. Samples with a ratio of 50% < S/N% ≤ 60% were considered doubtful.

### 2.5. Molecular Test

#### Real-Time RT-PCR

Total RNA was isolated from the collected blood samples and swabs using a kit (ID Gene™ Mag Fast Extraction Kit (IDvet, Grabels, France). Aliquots of RNA were stored in a freezer at a temperature of −70 °C until tested.

All types of samples were analyzed by real-time RT-PCR to detect the presence of viral nucleic acid with the RT-qPCR kit (ID Gene Tempeste des Petits Ruminants Duplex, IDvet genetics, Grabels, France), using the Applied Biosystems 7500 system (Carlsbad, CA, USA).

### 2.6. Evaluation of the Protective Effectiveness of Passive Immunity

When the first two newborn lambs reached the age of 14 weeks, they were used for research to assess the effectiveness of passive immunity. Before starting the study, the lambs’ blood sera were tested for the presence of maternal antibodies in c-ELISA. The titer of maternal antibodies circulating in the blood sera of these lambs was 1:8. One of them was subcutaneously immunized with a vaccine at a dose of 1.0 × 10^3^ TCID50/mL. On 0, 7, and 14 dpv, blood samples were collected to determine the titer of serum antibodies.

The second 14-week-old lamb was used to assess protective passive immunity.

As unvaccinated animals, 2 lambs of 14 weeks of age were used, born first from unvaccinated ewes.

### 2.7. Challenge Study

On 14 dpv, 2 experimental lambs (one unvaccinated and one vaccinated) born with maternal antibodies and 2 unvaccinated lambs were inoculated with the control strain of PPRV (Kentau-7) [15] subcutaneously into the subscapular region in a volume of 1.0 mL (10^5.0^ TCID50). After control infection, experimental and unvaccinated animals were observed for 14 days with daily measurement of body temperature, sampling of swabs (nasal, ocular, oral and rectal) and blood, as well as the identification of clinical signs of PPR. Clinical signs of PPR detected in all animals were evaluated in points [16,17]. During the challenge study, severely affected lambs were withdrawn from the study and humanely euthanized.

### 2.8. Data Analysis

During the statistical analysis, GraphPad Prism software (version 8.0.1.) was used. Differences between antibody titers and between temperature and clinical parameters of experimental and control animals after immunization and infection with a control virus were determined using bilateral ANOVA tests. The value of *p* ≤ 0.05 indicated that there was a significant difference between the data obtained. The difference in efficiency between the groups was compared using a one-sided Fisher exact criterion for two proportions at an alpha level of <0.05.

## 3. Results

### 3.1. Adverse Reaction Monitoring

**Figure 1:**
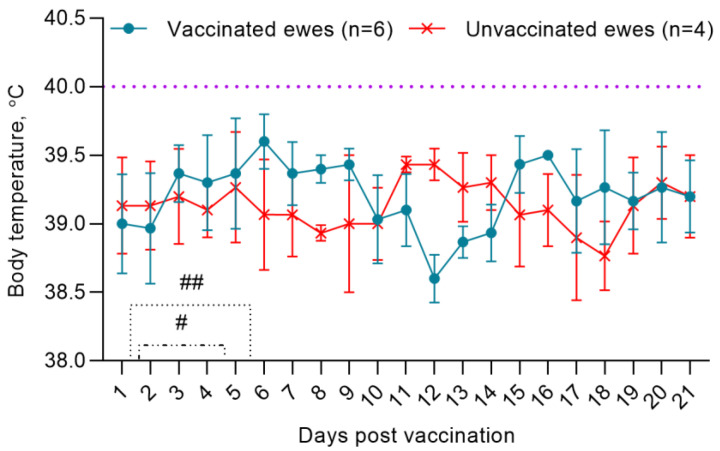
Dynamics of changes in rectal temperature of vaccinated and unvaccinated ewes. (#), (##) Duration of local reactions that occurred in vaccinated ewes post vaccination.

Post vaccination, the physiological state of pregnant ewes was within normal limits. The pregnancies of the ewes proceeded without any complications and as a result, healthy lambs were born. On the 2nd dpv, two ewes had a local reaction in the form of swellings with a diameter of 0.6 cm^2^, which disappeared within 4–5 days. During the observation period (21 dpv), the body temperature of the ewes fluctuated in the range of 38.5–39.8 °C (Figure 1).

### 3.2. Post-Vaccination Titers of Neutralizing Antibodies to the PPRV in Pregnant Ewes

**Figure 2:**
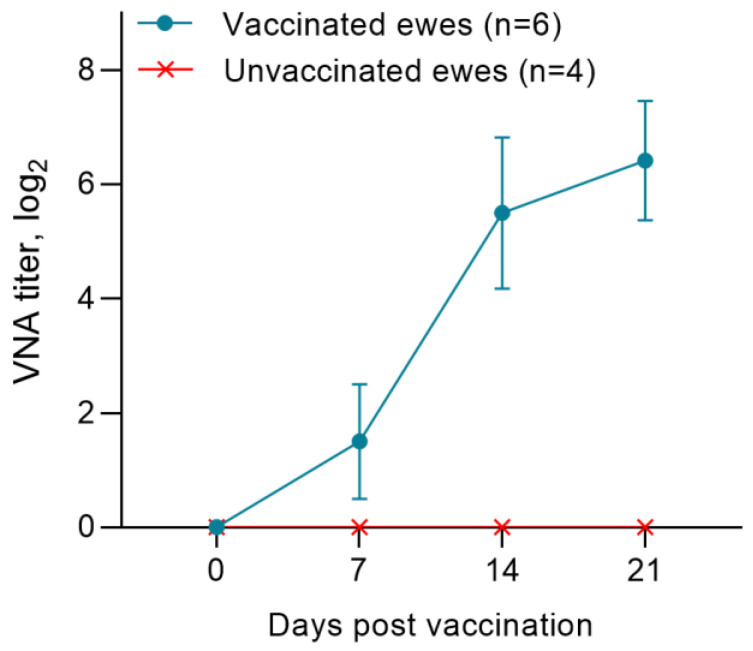
Accumulation of antibodies in ewes of the experimental and unvaccinated groups post immunization with the PPR vaccine.

Before vaccination (day 0), all pregnant ewes were seronegative for PPRV antibodies. In the first week post immunization, the average titers of neutralizing antibodies (NAs) increased to 1.6 log2. On 14 dpv, the level of NAs to the PPRV in pregnant ewes was 5.6 log2, and the indicated antibody titer increased to 7.2 log2 on the 21st day post vaccination (Figure 2). Unvaccinated ewes were seronegative for the PPRV (Figure 2).

### 3.3. Post-Vaccination Titers of Antibodies to the PPRV in ELISA in Pregnant Ewes

**Figure 3:**
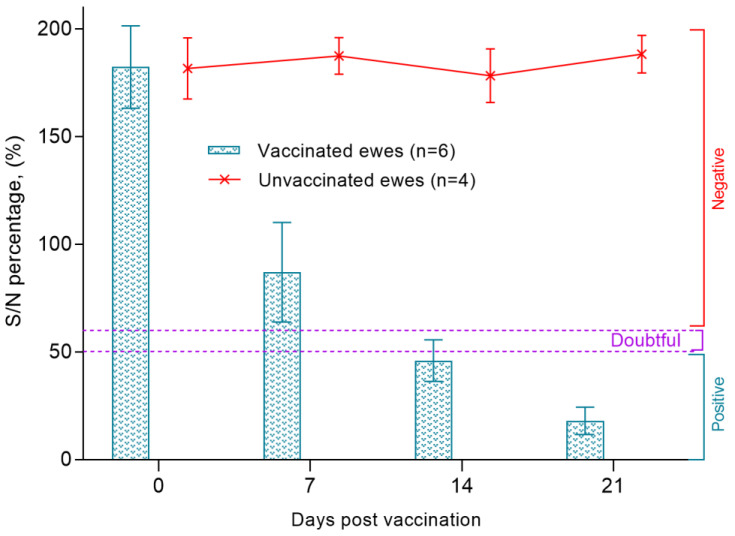
Antibody responses in ewes after vaccination against peste des petits ruminant virus, as measured by ELISA S/N ratio (%).

All samples of blood sera of pregnant ewes were negative before immunization with the vaccine against PPRV and had a ratio of S/N > 150%. Within 7 dpv, the S/N ratio in pregnant ewes was 87%. Protective titers of antibodies to the PPRV were found in 43% of animals. In 32% of animals, the titer of the produced antibodies exceeded the recommended threshold of S/N values (60%) for positive samples. In the remaining 25% of pregnant ewes, the results of c-ELISA were doubtful (50–60%). On 14 dpv, judging by the antibody titers, 92% of pregnant ewes were protected from PPRV, and the S/N ratio was 46%. The lowest S/N% value in vaccinated ewes (18%) was recorded on 21 dpv (Figure 3).

### 3.4. Titers of Maternal Antibodies in Lambs Born from Vaccinated Ewes

**Figure 4:**
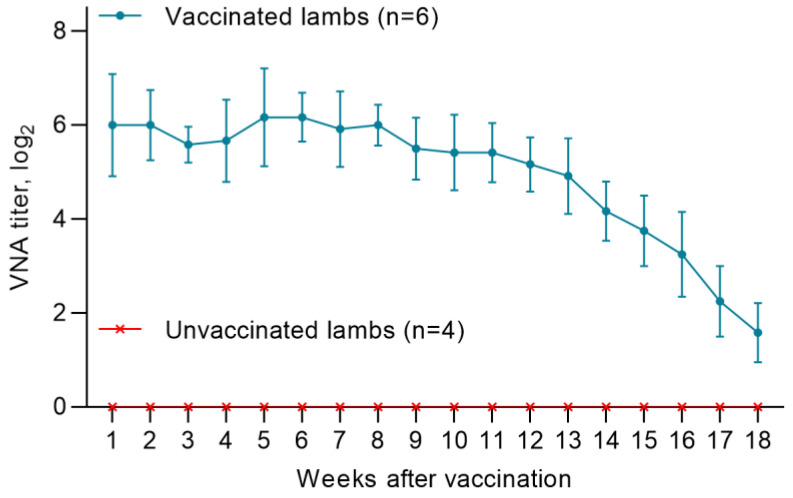
Dynamics of changes in the level of maternal antibodies in offspring born from vaccinated ewes.

A total of 10 lambs were born from vaccinated ewes at different times. In all lambs (n = 6) born from vaccinated pregnant ewes, the level of MAs produced was in the range of 5.5 log2 and 7.2 log2, and provided 100% humoral protection of the offspring from the PPRV up to the 14th week. Starting from the 14th week, there was a gradual decrease in MAs titers below the protective level (<1:8), and by the end of the 18th week, the MA titers in lambs were in the range from 1.0 to 2.25 log2 (Figure 4). Unvaccinated lambs (n = 4) were seronegative for the PPRV (Figure 4).

### 3.5. Titers of Antibodies to the PPRV in ELISA in Lambs Born from Vaccinated Ewes

Blood serum samples were collected from all lambs a week after birth to assess the level of MAs. In all collected samples of lambs’ blood serum, the S/N ratio was lower than <50%, which indicated a high level of passive immunity in newborn lambs.

**Figure 5:**
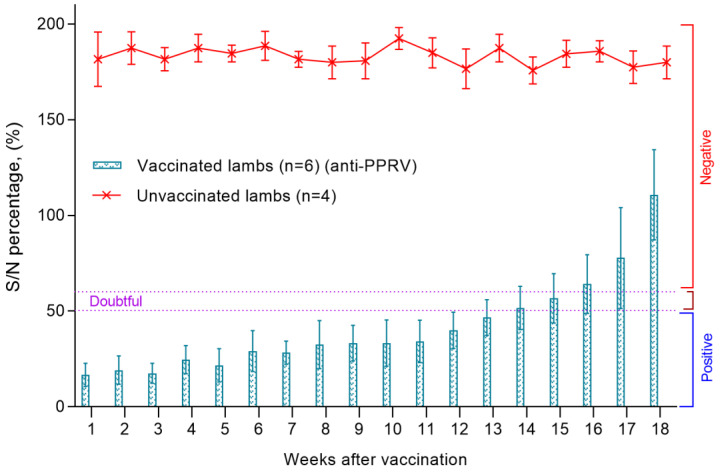
Assessment of the level of MAs reproduced in vaccinated and unvaccinated groups of lambs after birth from vaccinated ewes.

The level of MAs reproduced in kids persisted until the 14th week, while there was no significant difference between antibody titers (*p* > 0.05). At the same time, the average value of S/N was in the range of 20–55% (Figure 5). The blood sera of unvaccinated lambs were negative, since all samples had a ratio of S/N > 150% (Figure 5). From the 14th week, there was a gradual increase in the S/N ratio, which increased to 150% at the end of the experiment (week 18). Accordingly, by the end of the experiment, the level of produced antibodies in all lambs exceeded >60%, which indicated a decrease in MA titers in lambs below the protective level. The serum samples of the unvaccinated lambs were negative, since all samples had an S/N ratio >150% (Figure 5).

### 3.6. Post-Vaccination Titer of Neutralizing Antibodies to the PPRV in a Lamb Born with MAs

Before vaccination (day 0), the titer of NAs in the vaccinated lamb was 4.0 log2. In the first week post vaccination, the titer of NAs increased to 4.7 log2. At 2 weeks post vaccination, the level of NAs was 5.4 log2. PPRV antibodies were not found in the blood of the unvaccinated lambs.

### 3.7. Assessment of Viral Genomic Load in Blood and Swabs in Lambs

**Figure 6:**
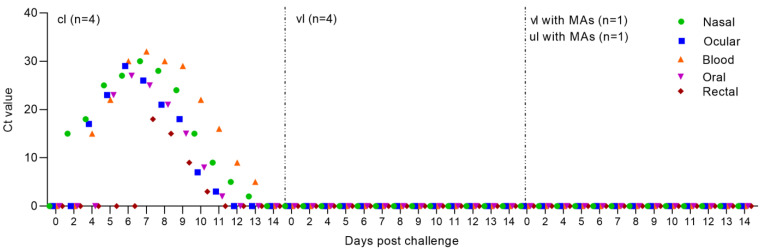
Quantitative determination of viral nucleic acid in blood samples and swabs of vaccinated and unvaccinated lambs post challenge. PPRV-specific RNA was measured by real-time reverse transcription PCR (RT-qPCR), and the amount of viral RNA is expressed as a Ct, a value that increases as the amount of viral RNA increases. Abbreviations: cl, challenged lambs; vl, vaccinated lambs.; vl with MAs, vaccinated lamb born with MAs; ul with MAs, unvaccinated lamb born with MAs.

PPRV genome was not detected in blood samples and smears of lambs born with MAs (Figure 6). Similar results were obtained when testing all types of samples collected from 14-week-old lambs (one vaccinated and one unvaccinated), used to assess the protective effectiveness of passive immunity (Figure 6).

In contrast, according to the results of RT-qPCR analysis, all types of samples taken from challenged lambs turned out to be positive (Figure 6). In challenged lambs, PPRV genome was detected from 2–3 days in nasal swabs, from 4–5 days in blood and ocular swabs, from 5–6 days in oral swabs and from 6–7 days in rectal swabs.

### 3.8. Evaluation of the Resistance of Experimental and Unvaccinated Lambs against Inoculation with the Field PPRV

**Figure 7:**
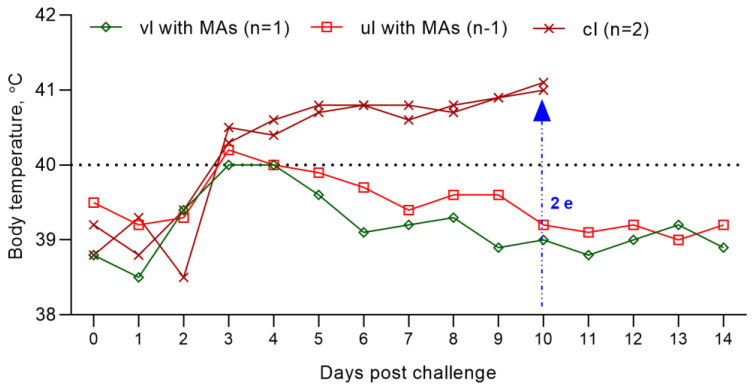
Dynamics of rectal temperature changes in lambs of the experimental (14-week-old lambs born with MAs (one unvaccinated and one vaccinated)) and unvaccinated groups after inoculation with the field strain PPRV. The graph shows the temperature reactions of one vaccinated, one unvaccinated (14-week-old lambs born with MAs) and two unvaccinated lambs post challenge. (2 e) On the 10th dpc, two unvaccinated lambs were humanely euthanized. Abbreviations: vl with MAs, vaccinated lamb born with MAs; ul with MAs, unvaccinated lamb born with MAs; cl, challenged lambs.

**Figure 8:**
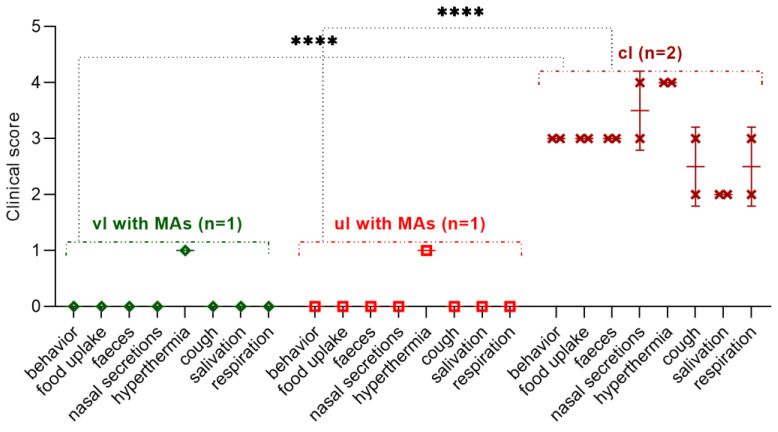
Manifestation of clinical signs in experimental (14-week-old lambs born with MAs (one unvaccinated and one vaccinated)) and unvaccinated groups of lambs after challenge with the strain Kentau-7. The graph shows the clinical scores of one vaccinated, one unvaccinated (14-week-old lambs born with MAs) and two unvaccinated lambs post challenge. Abbreviations: vl with MAs, vaccinated lamb born with MAs; ul with MAs, unvaccinated lamb born with MAs; cl, challenged lambs. ****, The difference between total clinical scores in experimental and challenged lambs.

Vaccinated at 14 weeks of age, the lamb was not ill with the manifestation of clinical signs of the PPRV. However, within 2 days (on the 3rd–4th day), he had an increase in body temperature to 40.0 °C, which then normalized (Figure 7); at the same time, the level of clinical indicators increased to 1 point (Figure 8). Also, the lamb had pink spot of irregular rounded shape with a diameter of 0.2 cm^2^ at the injection site of the vaccine, which resolved within 4 dpc. However, the results of the RT-qPCR analysis were negative.

An unvaccinated 14-week-old lamb born with MAs post challenge with the control strain had an increase in rectal temperature to 40.2 °C on the 3rd dpc, which normalized on the 6th day of the control test; at this stage (Figure 7), the lamb’s clinical score reached 1 (Figure 8). Also, a pink swelling of irregular rounded shape with a diameter of 0.3 cm^2^ was observed in the lamb at the injection site of the vaccine, which resolved within 6 dpc. All types of samples and swabs collected from an unvaccinated lamb as a result of PCR analysis were negative.

On 3 dpc, challenged lambs had an increase in rectal temperature, which reached up to 40.8 °C on 5 dpc (Figure 7). Pyrexia in infected lambs lasted for 8 days. On the 10th day of the control trial, pyrexia was observed in lambs equal to a clinical score of 4 (41.1 °C) (Figure 7 and Figure 8).

**Figure 9:**
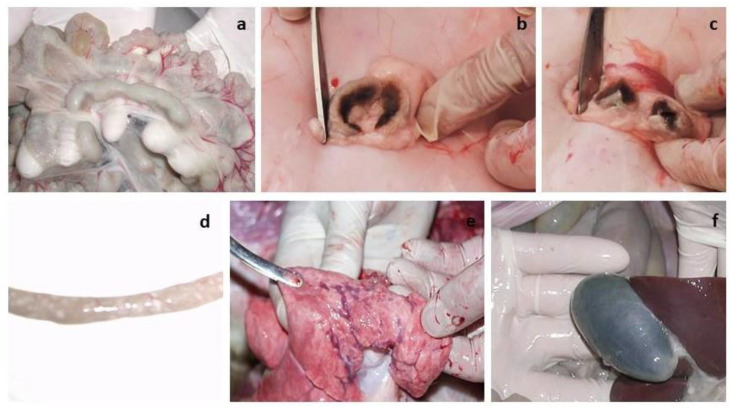
Pathoanatomic examination of euthanized lambs. (**a**–**c**) Enlarged lymph nodes. (**d**) pathological changes in the intestinal walls of euthanized lambs. (**e**) Hemorrhages under the serous membrane of the lungs. (**f**) A filled gallbladder.

In addition, the challenged lambs had clinical signs characteristic of PPRV (liquid transparent discharge from the eyes and nose), and a pink spot with a diameter of 2.0 cm^2^ was observed at the site of vaccine administration. On the 5th–6th dpc in intact lambs, nasal discharge became thick, purulent and yellow-greenish in color. In both challenged lambs, the gums became hyperemic. On the 7th–8th dpc wheezing was heard in the breasts of the lambs, while they had no appetite and were apathetic and sluggish, and liquid pus accumulated in the corners of the eyes (acute conjunctivitis). In addition, both lambs had loose stools, estimated at 3 clinical points (Figure 8). At 10 dpc, due to the deterioration in the general physiological condition of both challenged lambs, a decision was made on immediate humane euthanasia. The total clinical score in intact lambs before euthanasia was 41 points (Figure 8). After euthanasia, both lambs were subjected to necropsy (Figure 9).

### 3.9. Necropsy of Euthanized Lambs

During the necropsy and examination of the internal organs of the euthanized lambs, changes were found in the lymph nodes (mesenteric, pre-scapular, patellar), small intestine and lungs. The lymph nodes were enlarged (Figure 9a–c). Catarrhal inflammation and hemorrhages were observed in the mucous membrane of the small intestine (Figure 9d). Multiple dark red hemorrhages were observed under the pleura of the lungs (Figure 9e). The gallbladder was filled (Figure 9f).

## 4. Discussion

It is known that proper vaccination of ewes before mating is of great importance for the immune system of the offspring, since vaccines stimulate the production of MAs, which then pass into colostrum and provide newborn lambs with additional passive protection.

Conversely, vaccination of newborn lambs causes an insignificant immune response, because their immune system is not yet fully developed and cannot produce antibodies until the third or fourth week of life. In addition, it is known that vaccines administered to lambs up to 2 weeks of age bind MAs, and the young body remains defenseless.

Therefore, determining the optimal age for vaccination of young animals, which will come at a time when the level of MAs will significantly decrease and the young body will be able to develop its own protective adaptive immunity in response to the introduction of the vaccine with the formation of immunological memory, is of great importance. It should be noted that the formation of maternal immunity in offspring largely depends on the breed of the animal and the effectiveness of the strain used in the manufacture of a vaccine applicable for the immunization of pregnant animals.

Previously, in Kazakhstan, a live attenuated vaccine from the G45-MK strain was used to prevent PPR, which protected immunized animals from PPR for 1 year. Due to the short duration of immunity in vaccinated animals after immunization with a vaccine against PPR from the G-45MK strain, RIBSP employees decided to use a highly immunogenic strain (Nigeria 75/1) as the main agent for the development of a vaccine against PPR.

Although the Nigeria 75/1 strain is the main agent recommended by the OIE for the development of a vaccine against PPR, there is currently limited information on the optimal age at which lambs should be vaccinated with this vaccine.

These studies were devoted to assessing the duration of maternal immunity in lambs born from pregnant ewes of Kazakh breed fine-fleeced, immunized with a vaccine from the attenuated Nigeria 75/1 strain.

The post-vaccination results for pregnant ewes obtained by us proved the effectiveness of the vaccine used for sheep of the Kazakh breed fine-fleeced, since immunized pregnant ewes had protective antibody titers formed on day 14 (SN ≥ 1:8) to the PPRV (Figure 2 and Figure 3). In previous studies, the authors reported that protective antibodies to the PPRV were formed in Kano brown goats a week post vaccination with a similar vaccine [18]. This difference in the periods for formation of protective antibody titers is probably due to differences in the types of experimental animals.

Lambs born from sheep of Kazakh breed fine-fleeced immunized with a vaccine from the Nigeria 75/1 strain after receiving a sufficient amount of colostrum had protective levels of maternal antibodies against PPR for 14 weeks. From week 14 to the end of the experiment (18 weeks), a decrease in antibody activity was observed, and at week 18, all lambs had titers <1:8 (Figure 4 and Figure 5). In this connection, lambs of the Kazakh breed fine-fleeced are proposed to be immunized at the age of 14 weeks or older, when the maternal immunity is sufficiently weakened and the lamb can develop its own immune response to the introduction of the vaccine.

Similar results were reported in a previously published work by Bodo et al. (2006), where the authors recommend immunizing lambs born from Djallonké ewes in the interval from 11 to 14 weeks after birth [19].

Markus et al. (2019) and Abdollahi et al. (2023), who conducted similar studies, recommend immunizing kids born from Kano brown and Saanen goats vaccinated with the Nigeria 75/1 strain at the age of 10 weeks [18,20], while Olushola S. Olaolu et al. (2021) suggest vaccinating Yankasa lambs at the age of 9 weeks [21]. In these studies, differences in the age at which kids should be vaccinated may be related to the types and breeds of experimental animals used in the studies. It has previously been proven that the same PPR vaccine can cause different immune responses in different breeds of goats [22].

It is known that for reliable protection of animals from PPR, the level of VNA in the blood serum of animals should be ≥1:8 [23,24]. Since the lambs were born at different dates, two lambs of 14 weeks of age with a serum antibody titer of 1:8, born first from vaccinated ewes, and two lambs of the same age, born first from unvaccinated ewes, were used to evaluate the effectiveness of passive immunity in lambs. At the same time, in the blood serum of the vaccinated lamb, the antibody titer increased above 1:16 for 14 days, and the lamb was 100% protected from the control virus (Figure 7). An unvaccinated lamb of 14 weeks of age, born with maternal antibodies, was also fully protected during active infection with the control PPRV (Figure 7).

In a similar study conducted by Balamurugan et al. (2012) in a lamb immunized with the Sungri vaccine strain, protective titers of antibodies to the PPRV began to increase from the 21st day post vaccination (21 dpv) [11]. It is possible that the difference in the period of the formation of immunity in kids in these similar studies is due to the difference in the vaccine strains used in the two live vaccines, and also, the possible influence of the type and breed of experimental animals on the obtained research results is not excluded.

The total clinical score calculated by us during the control trial using the field strain PPRV was for the experimental and control groups of lambs 2 and 41 (Figure 8), respectively.

At the same time, the lambs of the experimental group had a two-day increase in body temperature and pink spots on the site of the introduction of the vaccine, which disappeared by themselves within 4 dpc (Figure 7).

However, the results of RT-qPCR analysis turned out to be negative when examining blood samples and swabs collected from an experimental group of lambs after vaccination and control infection (Figure 6).

The unvaccinated lambs showed pronounced clinical signs of PPRV after the control test. In addition, all swabs and blood samples collected from the unvaccinated group of lambs were positive when examined using RT-qPCR (Figure 6). Due to the deterioration in their general condition, both lambs of the unvaccinated group were euthanized using an injectable drug belonging to the barbiturate group (Figure 7).

The results of this study once again prove the safety and effectiveness of the vaccine from the Nigeria 75/1 strain for pregnant ewes, since the vaccine did not affect the course of pregnancy in ewes of the Kazakh breed fine-fleeced, while all ewes were protected from PPR from 2 weeks post vaccination. The maternal immunity formed in lambs born from vaccinated sheep of the Kazakh breed fine-fleeced persisted until 14 weeks after birth, with slight fluctuations in antibody titers (*p* > 0.05). Although a minimum number of lambs were used to determine the effectiveness of the vaccine, the results obtained indicate that lambs born from a fine-fleeced Kazakh breed should be vaccinated from the age of 14 weeks or older.

It is important to note that, despite the limited number of animals in our study, the results offer opportunities for careful design, meticulous data analysis, and collaboration. By implementing these strategies and viewing this limitation as a stepping stone for future research, we can work toward eliminating this constraint and gaining more robust insights in the future.

## 5. Conclusions

Analyzing the results we obtained in this study, we came to the conclusion that during routine vaccination against PPR, lambs born from sheep of Kazakh breed fine-fleeced should be immunized from the age of 14 weeks or older to avoid a period of susceptibility in lambs to the PPRV. Since the same vaccine against PPR can cause different immune reactions in different breeds of sheep and goats, it is necessary to conduct further studies on other breeds of sheep and goats living in Kazakhstan in order to determine the appropriate period of immunization of small cattle with a vaccine from the Nigeria 75/1 strain.

## Data Availability

Not applicable.
